# “Don't let anybody ever put you down culturally…. it's not good…”: Creating spaces for Blak women's healing

**DOI:** 10.1002/ajcp.12607

**Published:** 2022-08-01

**Authors:** Paola Balla, Karen Jackson, Amy F Quayle, Christopher C Sonn, Rowena K Price

**Affiliations:** ^1^ Moondani Balluk Victoria University Melbourne Victoria Australia; ^2^ Indigenous, Moondani Balluk Victoria University Melbourne Victoria Australia; ^3^ Institute for Health and Sport Victoria University Melbourne Victoria Australia; ^4^ Moondani Balluk and Institute for Health and Sport Victoria University Melbourne Victoria Australia; ^5^ University of the Witwatersrand Johannesburg South Africa; ^6^ Eternal Earth Connections Melbourne Victoria Australia

**Keywords:** bush dyeing, Country, cultural practice, healing, Indigenous knowledge, Wayapa Wuurrk

## Abstract

Research has highlighted the importance of Indigenous knowledge and cultural practice in healing from ongoing histories of trauma, dispossession, and displacement for Indigenous peoples in Australia and elsewhere. Connection with culture, Country, and kinship has been identified as protective factors for Aboriginal social and emotional well‐being and as facilitating cultural healing. This paper draws on stories mediated through cultural practice specifically, Wayapa and bush‐dyeing workshops, to explore how women resignified experiences and engaged in “healing work.” Our collaborative analysis of the stories shared resulted in three main themes that capture dialogs about the need for culturally safe spaces, vulnerability and identity, and culture, Country, and place. Centering Aboriginal knowledge, our analysis shows the meanings of Country, spirituality, and the coconstitution of people, culture, and the natural environment. Through Indigenous cultural practice, the women “grew strength in relationship” as they engaged in the psychosocial processes of deconstruction, reclamation, and renarrating personal and cultural identities.

## Highlights

- Aboriginal communities continue to experience historical trauma and ongoing structural violence.
- Aboriginal people have expressed the need to understand the impacts of colonial dispossession.
- Unpacking systems of oppression and cultural reclamation is central to decolonization and healing.
- Embodied cultural practices allow Aboriginal women to yarn about culture and Country.
- Relationship building is central to empowering inquiry as healing.

## INTRODUCTION

The deleterious psychological, social, and cultural effects of colonization, patriarchy, and other systems of domination have been well documented in various bodies of literature (e.g., Bulhan, 1985) and various inquiries (Aboriginal and Torres Strait Islander Healing Foundation, 2017; Human Rights and Equal Opportunity Commission, 1997). In Australia, a central part of the grief and trauma experience for Aboriginal people has been the systematic dispossession of culture, family, and ancestry through assimilationist policies and practices including the systematic forcible removal of children from their families (Haebich, 2000). In Australia, Judy Atkinson (2002) wrote about psychosocial domination or cultural genocide as a form of violence used to control Aboriginal and Torres Strait Islander peoples, along with direct and structural violence:

Cultural genocide not only works to destroy the cultures of oppressed peoples, it also eradicates the sense of worth, of self‐worth, and of well‐being in individuals and groups so that they are unable to function from either their own cultural relatedness, or from the culture of the oppressors. They feel in a world between, devalued, and devaluing who they are. (p. 71)

This points to the propensity for oppression to be internalized, fracturing families and communities and across generations (David, 2014; Gonzalez et al., 2014). Indeed, complex issues affecting Indigenous individuals, families, and communities have been understood as stemming from what has been variously described as collective, historical, and intergenerational trauma (e.g., Duran et al., 1998; Kirmayer et al., 2014; Menzies, 2019). As noted by Kirmayer et al. (2014), the concept of historical trauma has been useful in highlighting the ongoing impacts on Indigenous individuals and communities of “colonization, marginalization, and cultural oppression” (p. 313). Importantly though, the authors emphasized that the persistent suffering of Indigenous peoples reflects not only past trauma but ongoing structural and symbolic violence.

In the west of Melbourne, the unceded lands of the Boonwurrung and Woiwurrung of the Kulin Nation, in which we live and/or work, there are many Aboriginal families with unknown language group identity and connection to Country with many apart of or descendants of the Stolen Generations (Jackson, 2019). In this context, Aboriginal people have expressed a need for culturally safe spaces, a desire to understand the impact of dispossession and dispersal on their identity and community connection and for opportunities to renew connection with culture, Country, and kinship (Balla, 2020; Sonn et al., 2018). In their study on place‐making by Aboriginal people in Melbourne's west, Sonn et al. (2018) reported stories of the effects of dispossession including policies and practices of child removal, and how this continues in the lives of people in the present. They also reported on policies and programs that are being put in place to respond to the needs of Aboriginal people and the vital role of research and action that can advance the goals of decolonization, empowerment, and promoting individual and community wellbeing. While there are diverse pathways to achieve these goals, one area has involved the creation of culturally safe spaces in which people can deepen their knowledge of culture, history, and community, unpack systems of oppression, and create, renew, and reclaim cultural practices that are central to decolonization and healing.

The concept of cultural safety originated in Aotearoa/New Zealand in response to the health inequity experienced by Maori people. The term refers to:

[A]n environment that is safe for people: where there is no assault, challenge or denial of their identity, of who they are and what they need. It is about shared respect, shared meaning, shared knowledge and experience of learning, living and working together with dignity and truly listening. (Williams, 1999, p. 213)

Cultural safety is expressed in various policy frameworks in the Australian context, particularly in relation to mainstream service delivery but also within Aboriginal communities as an Aboriginal culturally informed tool “to ensure that Aboriginal community environments are also culturally safe and promote the strengthening of culture” (Frankland et al., 2011, p. 27). Cultural safety involves “working to enhance rather than diminish individual and collective cultural identities, and empower and promote individual, family and community wellbeing” and “is crucial in enhancing individual and collective empowerment and more effective and meaningful pathways to Aboriginal self determination” (Walker et al., 2014, p. 201).

The project reported in this paper looked to provide culturally safe spaces for Aboriginal women to come together to talk about culture, Country, and connections mediated by embodied cultural practices. This knowledge could also be embedded in their daily life. Informed by Indigenous knowledge systems and Aboriginal‐led inquiry, we sought to document and explore the “healing work” that is done through Aboriginal self‐determined cultural workshops.

## CONCEPTUAL FRAMEWORK: CULTURALLY SAFE SPACES AND COUNTERSPACES

The recovery and reclamation of Indigenous knowledge systems, the renewal of cultural practices, and the creation of counter and culturally safe spaces have been identified as key strategies for decolonization that can strengthen community cultural identity, wellbeing, and facilitate collective healing (e.g., Aboriginal and Torres Strait Islander Healing Foundation, 2017; Archibald, 2006; Daher et al., 2020; Gone, 2016). The reclamation of culture is central to processes of reconstruction (Gone, 2016). For Rix et al. (2019) culture “is not just dance, ceremony, and language; it is a philosophy and worldview that is based on collective ways of knowing and being. Collective community is the relationship to all things from wind and weather to waters, land, and its people's” (p. 9). In Australia, the significance of Indigenous Knowledge Systems is reflected in the conception of Social and Emotional Wellbeing (SEWB) and of Country and its significance to SEWB. These notions conceived within an Indigenous worldview are central to Aboriginal practices of decolonization, recovery, community, and healing. Dudgeon and Walker (2015) noted that: Indigenous SEWB is complex and multidimensional and is situated within a framework that acknowledges “Indigenous Australian world‐views and expressions of culture, including the individual self, family, kin, community, traditional lands, ancestors, and the spiritual dimensions of existence …” (p. 278). Moreton‐Robinson (2003) noted that country is constitutive of Aboriginal people. Nonindigenous scholar, Deborah Bird Rose (1996) wrote:

Country is a place that gives and receives life. Not just imagined or represented, it is lived in and lived with. People talk about country in the same way that they would talk about a person; they speak to the country, sing to country, visit country, worry about, feel sorry for country, and long for country… (p. 7).

Connection with culture, Country, and kinship has been identified as protective factors for Aboriginal SEWB (Zubrick et al., 2014) or as cultural determinants of health (Verbunt et al., 2021) and reconnecting to Country, family, and culture as a means of facilitating cultural healing (Rix et al., 2019).

As asserted by Wilson (2004), Indigenous knowledge recovery as an anticolonial project is Indigenous empowerment because it is a “conscious and systematic effort to revalue that which has been denigrated and revive that which has been destroyed. It is about regaining…ways of being” (p. 359). Fredericks (2010) noted that re‐empowerment for Aboriginal women necessarily entails “rebuilding and reviving indigenous women's spiritual and cultural practices accompanied by healing” (p. 548) and is reflected in Aboriginal people “naming what Aboriginal identity is to them, how it manifests itself, and the experiences, rights, roles, and responsibilities of Aboriginality” (p. 548). For Fredericks (2010), “this form of articulating is part of Aboriginal women's resistance to the ongoing processes and impacts of colonization and is part of our reempowering of ourselves as Aboriginal women” (p. 548). The creation of counterspaces is central to liberatory practice. Building on the concept of homeplace coined by Black feminists (see hooks, 1990), Case and Hunter (2012) proposed the counterspaces framework to conceptualize and investigate responses to oppression, and in particular, the person–environment transactions that foster adaptive responding. Within this framework, the role of “narrative identity work, acts of resistance, and direct relational transactions” (p. 262) are recognised as contributing to self‐enhancement within counterspaces. They described narrative identity work as involving the creation and maintenance of oppression narratives, resistance narratives, and reimagined personal narratives. Oppression narratives “affirm and privilege the individual's subjective experience of oppression” (p. 263); resistance narratives, articulate “the strength and capability of setting members to overcome oppression” (p. 264), and reimagined personal narratives, as an individual level construct, “re‐craft individual identities which have been mis‐represented and demeaned through dominant cultural narratives” (p. 263). Narrative identity work is also an interpersonal process with shared narratives functioning to strengthen the community and to foster the bonds of solidarity. Others have similarly written about the significant role of narratives in resistance and resilience processes (e.g., Quayle & Sonn, 2019; Rappaport, 1995; Sonn & Fisher, 1998). Based on their work with Aboriginal people in Canada, Kirmayer et al. (2011) noted that collective forms of narrative “help people make sense of their experience and construct a valued identity but also ensure the continuity and vitality of a community or a people” (Kirmayer et al., 2011, p. 86). Informed by Indigenous understandings of resilience and personhood and recognising the power of stories and storytelling, they discussed narrative resiliencies having

communal or collective dimension, maintained by the circulation of stories invested with cultural power and authority, which the individual and groups can use to articulate and assert their identity, affirm core values and attitudes needed to face challenges, and generate creative solutions to new predicaments. (Kirmayer et al., 2011, p. 86)

Counterspaces also provide members opportunities “to think, feel and act in ways that are consonant with their own identities but that are typically devalued by the larger society” (Case & Hunter, 2012, p. 265). This may involve enacting cultural practices including rituals, dress, and language, as well as an explicit critique of the oppressive conditions characterizing their lifeworlds. These practices of affirmation and critique are understood as acts of resistance that enhance the self‐concept of marginalized groups, foster individual and community identity, sense of community, solidarity, and support. Counterspace participants also benefit from direct relational transactions including social support and the social transmission of strategies to respond to the experience and consequences of oppression.

Critical and Indigenous scholars and practitioners have demonstrated how art and cultural practice produced in counterspaces can promote recovery of historical memory and counter‐memory, consciousness‐raising, witnessing, and healing (e.g., Martin & Mirraboopa, 2003; Quayle & Sonn, 2019; Segalo, 2013; Segalo, 2018; Távara et al., 2018; Watkins & Shulman, 2008). Aboriginal scholar, Atkinson (2002) in her *We Al‐li* program discussed the importance of creating culturally safe spaces for people to share their stories as part of the relational process of healing. Atkinson discussed the significance of recognising your story as part of a collective story. In the context of a digital storytelling project in Aotearoa, Baltrán and Begun (2014) similarly discussed the transformative power of storytelling in terms of people finding and sharing their story, of seeing their stories reflected in the stories of others, and of the supportive environment created through such projects, which fosters feelings of safety, freedom, and interconnectedness. Balla (2020), through practice‐led inquiry, guided by Aboriginal Feminist standpoint theory and sovereignty, witnessed and responded to women's art‐making and activism. Through a combination of community cultural practices including bush dyeing, Balla created healing cloths that materialized “acts of daily repair,” unconditional love, and restoration. Kasat (2020), in a series of case studies, demonstrated how arts practice can make visible various forms of violence impacting the lives of marginalized women and open opportunities for healing. C. C. Sonn et al. (2017) showed how cultural practice can foster hope and promote healing for Aboriginal communities suffering the devastating effects of violence and the impacts of suicide. They described the sensitive culturally anchored and reflexive process that facilitated community storytelling rooted in Aboriginal, Noongar experiences and knowledge. It was from the lived experiences of the Aboriginal women leading this group, and this growing body of literature that we sought to craft a decolonial project aimed at fostering Blak women's healing.

## RESPONDING TO THE NEEDS OF ABORIGINAL WOMEN: WAYAPA WUURK AND BUSH‐DYEING WORKSHOPS

Our project builds on strong relationships that we have forged through community‐making activities with Aboriginal groups in Melbourne's west and our research and teaching collaborations over more than 10 years between members of the team made up of Jackson (Yorta Yorta), Price (Yorta Yorta and Palawa), Balla (Wemba Wemba and Gunditjmara), Sonn (migrant of color to Australia from South Africa) and Quayle (white Australian). Our interdisciplinary work draws from critical, liberation and community psychology and Indigenous studies, and is specifically concerned with understanding and disrupting the dynamics of oppression and promoting liberation in the everyday lives of people and communities, practice, and research (Balla, 2020; Quayle & Sonn, 2019; Sonn & Quayle, 2012; Sonn et al., 2017). As nonindigenous colleagues, Sonn and Price are aware of their differential social locations, subject positions, and the need for critical reflexivity within the context of racialised and gendered power relationships that shape their efforts to support Aboriginal self‐determination and disrupt coloniality.

Through cultural workshops delivered by Aboriginal women, specifically Wayaapa Wuurk (connect to the earth) and bush dyeing, we sought to create a culturally safe space for Aboriginal women to come together to yarn about their lives, past, present, and future and strengthen their connection with culture and Country. In yarning about their lives and their connections with culture, Country, and each other, the aim was to support Aboriginal women to not only understand the impacts of colonial dispossession on identity and community connection but also to engage in “rebuilding and reviving indigenous women's spiritual and cultural practices” (Fredericks, 2010, p. 548) as central to healing. The series of workshops consisted of 10 Wayapa workshops followed by four bush‐dyeing workshops, with the same group of women invited to attend each of the workshops.

### Wayapa Wuurrk

Through a method of Earth mindfulness, storytelling, movement meditation, and taking action to look after Country, Wayapa aims to foster connectedness and belonging while supporting holistic well‐being. Within this cultural immersion program, a series of 14 elements are used to teach participants the importance of connecting to earth and nature for wellbeing. Wayapa Wuurrk enables the sharing of traditional and Aboriginal story and cultural practices related to place, Country, and landscape. This component was delivered by Price (Yorta Yorta and Palawa) of Eternal Earth Connections. Jackson is a licenced Wayapa Wuurrk practitioner who also draws on her training in family and systems therapy. The typical embodied process teaches people how to center their mind and body along with introducing language, skills, and resources to deconstruct and reconstruct understandings of self, Country and culture.

Originally, we had planned to deliver the sessions over 14 weeks at the Wunggurrwil Dhurrung Centre in Wyndham Vale. However, the extended lockdown thwarted our plans. We reset and price revised the plan to deliver weekly 2‐hour sessions over a 10‐week period using the zoom platform. The typical structure of each session included an Acknowledgement of Country, checking in, element discussion, deep listening, and next steps. Deep listening here is inspired by the notion of Dadirri as conceived by Mirian‐Rose Ungunmerr (1988). As noted by Ungunmerr (2017), “In our language this quality is called *dadirri*. It is inner, deep listening and quiet, still awareness” (no page number). While online delivery was not the preferred platform, permissions had recently been adapted to include online delivery to respond to the needs of individuals during the pandemic. Information collected from a postworkshop survey showed that most of the participants found that participation was a valuable experience, especially during lockdown; as reported by a participant, “it provided the women with some time for self‐care, and some others who had attended previous sessions felt that online was 'just as good'.”

### Bush dyeing

Bush dyeing is a creative art practice that uses indigenous local flora to make healing cloths. The bush‐dyeing workshops were led by Balla (Wemba‐Wemba and Gunditjmara woman). As noted by Balla (2020), the clothes are a form of journaling as they are about mark‐making. Creating the bush‐dyed fabrics, scarves or clothing is a way to respond to Country, trauma, memory, and give healing opportunities. Tunstall (2015) argued that bush dyeing provides an opportunity for people to re‐root themselves to culture and Country. The bush‐dyeing practice involves walking and being in local landscape and environments to collect bush materials and artifacts for use in workshops where the women make healing cloth pieces for display or to wear and yarn with other Aboriginal women about the process and potential outcomes, but also about everyday life as Aboriginal women including the need for healing and acknowledging traumas.

In her arts and healing practice, Balla (2020) has found that the bush‐dyeing process brings participants into a mindful state of being and doing as it is deliberate, careful, and thoughtful slow work. These meditative practices are both cultural practices of being with Country and with other Aboriginal Peoples, for women, in women's business, of yarning and listening and gathering. It also involves creating a space to be validated, heard, and to practice just being. Given COVID‐19 restrictions, the bush‐dyeing workshops also had to be delivered online. Four, two hour workshops were delivered where the women involved were introduced to the art of bush dyeing and provided instructions and a demonstration on how to do it and then came together to share what they had created each week and reflect on the process.

Each woman was gifted a bush‐dyeing kit, which included a large pot, calico, a silk scarf, long tongs, vinegar, string, a rusty object, and turmeric (for the color). Given COVID restrictions, each of the participants did their bush dyeing in their own time at home. The process involved the women collecting plants including gum leaves while walking in the Country. The collected plants are then laid out in a pattern, or laid out randomly before being wrapped up in a bundle to boil in a pot for one to three hours. Gum leaves go into the pot of boiling water, along with a rusty object to serve as a mordant to draw patterns and oils from the eucalypts and plants. The longer they sit, the stronger the marks and patterns become. The decision is up to each participant. Balla gave advice on her experiences of making the clothes, but emphasized that the decisions about the cloth belong to the cloth maker. A cultural “reading” process takes place with each cloth, as each one is completely distinct and unique to another. This is viewed as different spirits, of the plants, insects, animals, and Ancestors of the place the plant material comes from and appears on the surface of the cloths. After drying, the clothes are ironed. The hot iron “sets” the fabric and the images that have emerged. This is a laborious and involved process, and the repetition and attention to detail can be soothing and meditative. Being present is critical to this study, but so is being calm, and the work itself brings on a sense of timelessness and presence. The smells of the boiling pot emanate the air. The eucalyptus oil creates a sense of being in the bush after rain or by the campfire; it is evocative of the bush and being in the Country and it is comforting to work.

Both practitioners emphasize the mindfulness and tenderness of the healing work in these workshops and the importance of acknowledging traumas without triggering or exploiting trans‐generational traumas that are unresolved in families and communities and manifest in various ways. They stress reciprocity as a principle and practice central to the workshop process where cultural processes meet with inquiry and dialog.

## METHODOLOGY

Consistent with Indigenous approaches to knowledge production, image/object/idea led yarning and participant observation were the key methods of storytelling for this project (Kovach, 2018). Storytelling methodology is broader than just a data collection technique; it is a way of knowing that includes yarning and sharing of cultural resources that communicate meaning and through which people make meaning. Bessarab and Ng'andu (2010) have described yarning as “an Indigenous cultural form of conversation” (p. 37) “… conducive to an Indigenous way of doing things; its strength is in the cultural security that it creates for Indigenous people participating in research” (p. 47). Yarning is a process that requires the researcher to develop and build a relationship that is accountable to Indigenous people participating in the research” (Bessarab & Ng'andu, 2010; p. 38). Central to this approach is the emphasis placed on “forming relationships that are based on equal and respectful partnerships, support, cooperation and respect” (Fredericks et al., 2011, p. 16).

Typical in decolonizing approaches is that “… the spaces and communities that give life to these projects take precedent over questions of method and strategy” (Zavala, 2016, p. 66). Our project was framed through this approach and involved creating a space for Aboriginal women to engage in cultural, healing practices to support them to understand the impacts of colonial dispossession on identity and community connection. In line with decolonizing and Indigenous approaches (Dudgeon & Walker, 2015; Snow et al., 2016; Zavala, 2016), our broader commitment to generative and engaged praxis, the first step was to gauge the interest of women in cultural workshops, specifically Wayapa and bush dyeing.

### Methods

The project received ethics approval from the Victoria University Human Research Ethics Committee. Our project was to be delivered face to face, but because of the second lockdown to control the spread of COVID‐19, the team pivoted to deliver the program online. A total of 14 women took part in the workshops over the 3 months. Women were recruited via the community networks of Paola and Karen. Participants who expressed interest received an information pack containing an information and consent form, and an On Country pack that included a scented candle, scarf, and journal. While 14 women in total participated, attendance varied over the weeks of the workshops because people are busy, and they named zoom fatigue and the strain of the lockdown (Table 1).

**Table 1:** Participant demographics

Name	Age	Ancestral country/heritage
Jane	55–65	Yorta Yorta
Pearl	40–50	Wemba Wemba and Gundijmara
Mona	40–45	Yorta Yorta and Palawa
Carol	55–65	Waddawurrung/Gunditjmarra
Diane	50–60	Gunditjmarra/Bundjulung‐
Sophie	35–45	Wurundjeri
Sarah	50–55	Biripi/Worimi
Nelly	25–35	Palawa
Kate	25–35	Bunurrung
Merle	20–30	Gunditjmara and Ngarrindjeri
Zeta	20–25	Palawa
Tonie	45–55	Nurrunga
Cat	35–45	Palawa
Darlene	35–45	Unsure

All members of the research team attended the first workshop, which involved the Acknowledgement of the Country, an introduction to the workshops, the research team, roles and process, and the clarification of any questions participants had. Christopher attended the first two sessions to introduce himself, to listen, to get a sense of the workshops, and how best to support the project, and then only attended fortnightly research team meetings. The decision to stop attending the workshops stemmed from the researcher's recognition that his participation in the women's space for yarning was temporary and subject to the group's permission. This was clarified early on when members commented that he would need to leave when women's business is discussed. In the second session, as one of the participants entered the Zoom space clearly upset, one of the other women suggested that he leave the room, assertion of Aboriginal women's authority—as a part of Women's Business. This signalled to us that the women were already making this space their own, asserting their authority as part of women's business in space for care, nurturance, and space where they could be vulnerable.

In the first session and throughout the workshops, participants shared their motivations for participation. Typically, the yarns were spontaneous, but the research team also probed by asking questions that we had developed to promote deeper reflection on experiences in the workshop. In addition to this process, the women also created a closed conversation group on messenger to stay connected, share experiences, images, and other reflections about their engagement in the project and other aspects they wished to share. Documenting reflections using messenger served as a proxy for reflective writing that was planned as a mode of data collection and was particularly important given the extended period of lockdown, which in Melbourne at the time included not being able to leave home other than for four essential activities (see Gibson, 2020). All the workshops were recorded and transcribed. Research team members also took notes of the sessions. Participants were invited to attend an in‐person yarning session once the lockdown regulations were eased as an opportunity to meet in person and for the women to reflect on the program. That session was also recorded and transcribed.

#### Data analysis

We collected a large body of stories that lends itself to various types of analysis and re‐presentation. Consistent with our approach of Aboriginal storytelling, the analysis was informed by critical narrative approaches (Quayle & Sonn, 2019) to understanding personal identity, relationships, and community, and the dynamics of power and resistance in everyday experiences. We understood stories as cultural resources that people use to make sense of self and others, and relationships including place, Country, history, and community (Quayle, 2017; Rappaport, 2000; Sonn et al., 2013). Stories and narratives are read to understand psychosocial and political processes of oppression, resistance, and liberation. This examination entails exploring the oppressive stories and practices in people's lives and how they reconstruct these in and through intentional dialogs about the past and present, self, place, and Country.

The process of analysis was dialogical and iterative guided by principles of reciprocity and accountability. As a form of analyst triangulation (Miles & Huberman, 1994), two team members read the initial transcripts guided by the research questions, conceptions of healing, and the notes provided by research team members who attended weekly workshops. As part of our process of analysis, the preliminary insights were shared with other members of the team for discussion and further refinement to ensure consistency of the interpretations. Together, members of the group generated broader themes that illustrate experiences of participation. Together, the experiences reflect the processes through which the women were growing “strength in relationship,” through care and nurturance and awareness‐raising. Each theme is illustrated by various voices to represent the range of experiences and meanings that were shared over the weeks. In our accountability, we used member checking (Miles & Huberman, 1994) to our interpretations of the stories and resulting themes shared with the women who attended the final workshop and are in the process of drafting this article. We also shared video updates of the project with the women and sought their input, which served as a form of verification of our reading.

## FINDINGS: GROWING STRENGTH IN RELATIONSHIP

Our analysis highlights the healing story that resulted over the weeks of being together in the space created for yarning through the cultural workshops. We discuss this as “growing strength in relationship.” Within this broader theme, we identified three subthemes. The first theme “creating a safe place together in challenging times” captured the impact of the lockdown and appreciation of being able to safely share the experiences with other women. The second theme, dialogs of vulnerability and identity, point to vulnerability as strength and starting to be embraced in the journey of examining and understanding the ongoing impacts of historical trauma and the process of cultural identity reconstruction. The third theme, dialogs about Country, place, and culture, points to the sharing of knowledge about culture, country, and spirituality that is central to counter‐narratives and decolonizing identities.

### Creating a safe space together in challenging times

It was not surprising that the experiences and impacts of the lockdown were often discussed in the weekly sessions. The selection of excerpts below illustrates some of the challenges and the interconnectedness of home/work/community spheres, the impacts of disruption upon their everyday life, and coping resources. For example, two participants spoke of work/community connections and the social and emotional impacts of disruptions in these spheres. One participant spoke of how working from home can be lonely, but that they are thankful to be working given the context of many people losing their jobs during the lockdown. She noted:

… I really am still very thankful for, you know, what I have. I still have employment, I still get a regular wage. I'm going okay. I do sometimes get a bit lonely, I live on my own and, um… You know, I've had some really busy workdays where I think, “Oh, this is just… This stuff they got me doing is just shit, it's, no.” (Diane).

Another person commented on community responses and the helplessness that she felt in her role, and how the lockdown intensified “unpleasant stuff” in work and community spaces. She expressed the emotional toll of community and work and how COVID‐19 was impacting in various settings:

Well, I actually wasn't going to come today cause, tonight, cause I'm, I'm very broken. So. Um, so, I've been crying. And listening to you guys makes me cry more. Um, but I'm still glad that I came. Um, I'm just, I'm very tired, I'm, I'm sad because today I've had, um, work and community, it's been non‐stop on the phone and I'm just seeing some un‐, hearing some unpleasant stuff that's happening being in lockdown. And it's just really got to me today and, I just don't feel strong enough to deal with criticisms from my workplace. (Sophie)

In addition to impacts on work, community, and home spheres, people expressed concern about not being able to be together, to provide support and care for others. They also shared how they coped and stayed centered every day. Diane stated that:

I'm enjoying walking my dogs in the day. So, look, not… You know. Um, I feel okay, you know, I'm sad about, you know, deaths in our community and stuff like that and we can't be together and, and talking to people about that, but, you know, it's just… Hopefully, I don't know, I think people are just going to go crazy in Victoria when they're allowed out. I think there's just going to be, you know, some great stuff happening, but there'll also be some really, like, disturbing stuff that's gonna happen, cause people are gonna go crazy and I don't know, yeah. (Diane)

Another person also highlighted the difficulties of being separated:

It's very difficult because I've got a lot of aunties who are very old and very fragile so, um, you know, um I would like to see 'em. You know, like if god forbid something happens, especially now with you know, Corona. I know they say it's a bit safe, but you know, I'm thinkin' oh, I get a phone call, what's happening, is everybody okay? So that, all, every time you receive a phone call from S.A. or one of my cousins, I'm thinking, is it good news or bad news. So you're sitting like, hoping everything will be okay. (Tonie)

These excerpts are illustrative of how taken for granted social relationships and support systems were disrupted, and the emotional toll of being isolated.

The workshops provided a safe space for people to gather. People described safety in different ways. Early on, when asked to describe their experiences of the workshops in one word one person said:

So, mine is warmth. I just get so much warmth from coming into this space. Um, and, and it's, um, sort of at it's, it's an open space, and there's trust, we have trust here. And I, and I think that's pretty amazing from, 'cause I was sorta worried about that at the start, like, how, like, a group of people that not everybody knew each other, you know, how, how, I thought it would take quite a while before, you know, people showed their emotions, and shared the sorta stories that made them cry. (Jane)

For others space meant connection with new people, history, and culture. For me:

the word for me would probably be connection. Um, I really haven't been a part of anything to do with my Indigenous heritage. Um, probably was the first person in the family to bring anyone really into it. Um, but yeah, I'm learning a lot. (Zeta)

Kate said:

Um, I wanna say it's been quite intimate, which is an interesting word of choice (laughs), but yeah. As I think I said last time, I was, like, every time it's like a, more a vulnerable space, I think. Um, so that's probably why I'd probably choose the word intimate. (Kate)

### Dialogs of vulnerability and identity

Both the workshops used Aboriginal storytelling and cultural practices. The settings were intentional, dialogical, and provided an opportunity for the women to explore knowledge about community, spirituality, and resources that they could explore in their homeplaces. Even though the sessions were delivered via zoom, the women commented that the workshops and the process made them feel safe and protected. Unlike traditional workshops, Wayapa‐ and bush‐dyeing centers Aboriginal relationality and spirituality and introduced to the women, knowledge and practices for self‐care, as well as knowledge about Country and culture. Younger women opened up and shared their experiences about identity and identification and the desire to learn more about being in the Country. They moved from feeling vulnerable to embracing vulnerability as affirming and vital to healing. One speaker noted about her journey:

But I just realised, like, it's, it's been my way to heal, by letting myself be vulnerable even though that makes you a target for people sometimes. I think it's more worth it because it helps other people talk and heal, and… I didn't know that before, I've started to learn that now. (Pearl)

As the dialog progressed, others spoke of vulnerability and the effects of closing down, and the importance of embracing vulnerability in the process of truth‐telling about lives. Vulnerability is vital to personal and collective strength and healing:

Kate: Just dob me in, that's fine. Um, I actually will give one sentence. Um, there is strength in, um, vulnerability, and that's how I feel in this. So, thank you.

Jane: That's all right. So are you feeling as vulnerable now as you were at the very first one?

Kate: I think it's been different, um, aspects of vulnerability. I think at the start it was more, um, I guess 'cause I didn't grow up in culture and I've only been able to reconnect to it in the last few years and, um, embed myself more so in it. Um, being that I didn't grow up on my country or anything like that, and didn't, um, know about it until I was a teenager.

Jane: Mm‐hmm (affirmative).

Kate: Um, and yeah I think this process and us, um, connecting strong women and, you know, telling stories and of that experience as well, different, uh, also. Similar in the feelings and the emotions that have happened for us. I think that's definitely helped quite a lot.

Elders transmitted cultural knowledge of country, place, and history and shared knowledge about coping and surviving symbolic, lateral, and institutional violence. People felt safe in sharing and transmitting knowledge and wisdom about their life journeys. This process was described as intergenerational wrapping around. Vulnerability is reclaimed in the process of intergenerational wrapping, which recenters wholeness. Passing on cultural information and skills and understanding how others have navigated and continue to deal with coloniality and lateral violence, as well as the ongoing legacy of the forced removal of children from families, is part of the dialog that takes place in the safe space.

Some participants wanted to explore their family histories and through the process understand the complex and painful process of navigating or reconnecting with their Aboriginal heritage later in life. For example:

Um, so for me, as a child of a, uh, Stolen Generation n, um, member, um, my family's, my mother's, um, her siblings story is pretty well known as my uncle, my mom's baby brother is [Name]. So for me, I, I'd like a little, to explore a little bit about, maybe if anyone has some, you know, stolen generate, generation history in their family, or maybe family members that, um, have found out a little bit later in life that there's some aboriginal heritage in their family. How does that impact them going forward, you know, as young ones or old, even older people that might have only, uh, discovered aboriginal heritage later in life, and how do they, uh, I guess navigate through that, you know, self‐identification aboriginal. How do they, um, what's the experience being accepted by community as a woman, you know, trying to pass that information onto their children when they're still doing their own journey. (Diane)

Another participant reflected those journeys of healing involve recovery, reconnection, and support to restore what the violent processes of colonization and the removal of children from families have stripped:

…. with the young, you know, find their journey and staying connected and you know, making sure they stay connected and find the connection, you know, their belonging and their journey and making sure that they're, you know, find the right connection back home, you know, to go back home and find their journeys. So if we can be any help or support, you know, I used to work in Link Up previously so, let me know. (Mona)

Participants raised questions about how patriarchy and colonisation disempowered women and their ways of knowing. For example, Mona noted:

…, I think through the process of colonisation, I believe as women, we've learned not to trust our own intuition. Um, you know, we, I‐, well I've heard a few sort of references to, like, silly or weird or, um, and I think that itself is through colonization. Do you know what I mean? Like, we, um, b‐ before colonization we just, I don't know, would've known our role, would've known our responsibility, um, I believe we would've s‐ been a lot stauncher with each other.

In another sequence, a participant shared a story about becoming and about being proud and strong women, a process of learning with and affirming Aboriginal culture. It is about navigating oppression and creating an identity as a proud Aboriginal woman. This included deconstructing imposed categories for understanding self and ancestral heritage reclaiming:

Um, I went over to, um, New Zealand no long back, and I met with the First Nations women over there to discuss our uses on seaweed and done a comparison. And even though, um, they appear to be stronger in culture than us, we found that we had a better understanding of our inner self when we're allowed to feel it.

So, when you trust your own people to be with you and that the spiritual people, I believe you, that walk with us, that's when we become strong women.

And when other people, we done a thing with our clap sticks once, and this guy come up, and he was from India and they use big clap sticks in, they go, “We use them in ceremony.” We go, “So do ours.”

But it doesn't mean that their culture was stronger than my belief in my culture. So, all I can say to any woman that's on a journey of culture: *Trust your own instinct in culture and your growth in culture*. And never compare your growth in your culture to anybody else's culture, because no two cultures are the same. (Carol)

The women participating were at various stages in their identity journeys.

### Dialogs about country, place, and culture

Passing on cultural knowledge and ensuring continuity was evidenced in counter practices such as the delivery of the Welcome or Acknowledgment to County, the appropriation of Wayapa modalities, and creation of healing clothes using plants and other materials linked to the Country. While the acknowledgement is typically performed by an Elder, in one of the later sessions, another group member was invited to do this:

Hello. Elders, ancestors, and great spirits of the land I stand upon, I acknowledge you. I am a woman, and when I speak to my ancestors, they ask me to pass on the salutations to you, the traditional custodians of the Wathaurong land we meet upon. I pay respects to you and your elders, present and emerging. I pay respect to your laws, customs, traditions, and living cultures, which have existed since time forever. I extend that respect to all First Nations people throughout this land, some who are here today. And may we meet and have conversations as friends who respect and care for each other. (Sarah)

In another example, one facilitator conveyed a story of cultural knowledge and the interconnectedness between place and community. These dialogs are restorative and affirmative of ways of knowing and being that reinforce relationship to place:

Yep like Aunt said, please do some gathering of gum leaves and bush flowers today or tomorrow before 6.30. We will do a dyeing run through tomorrow night. In my Mob Wemba‐Wemba we are not allowed to bring wattle in the house. My Nan was very strict on it because it was about mourning time‐wattle was only to be put on graves. Not all Mob do though‐like with willy wagtail who is a good sign for us/but sign of warning for other Mobs. (Pearl)

**Figure 1:**
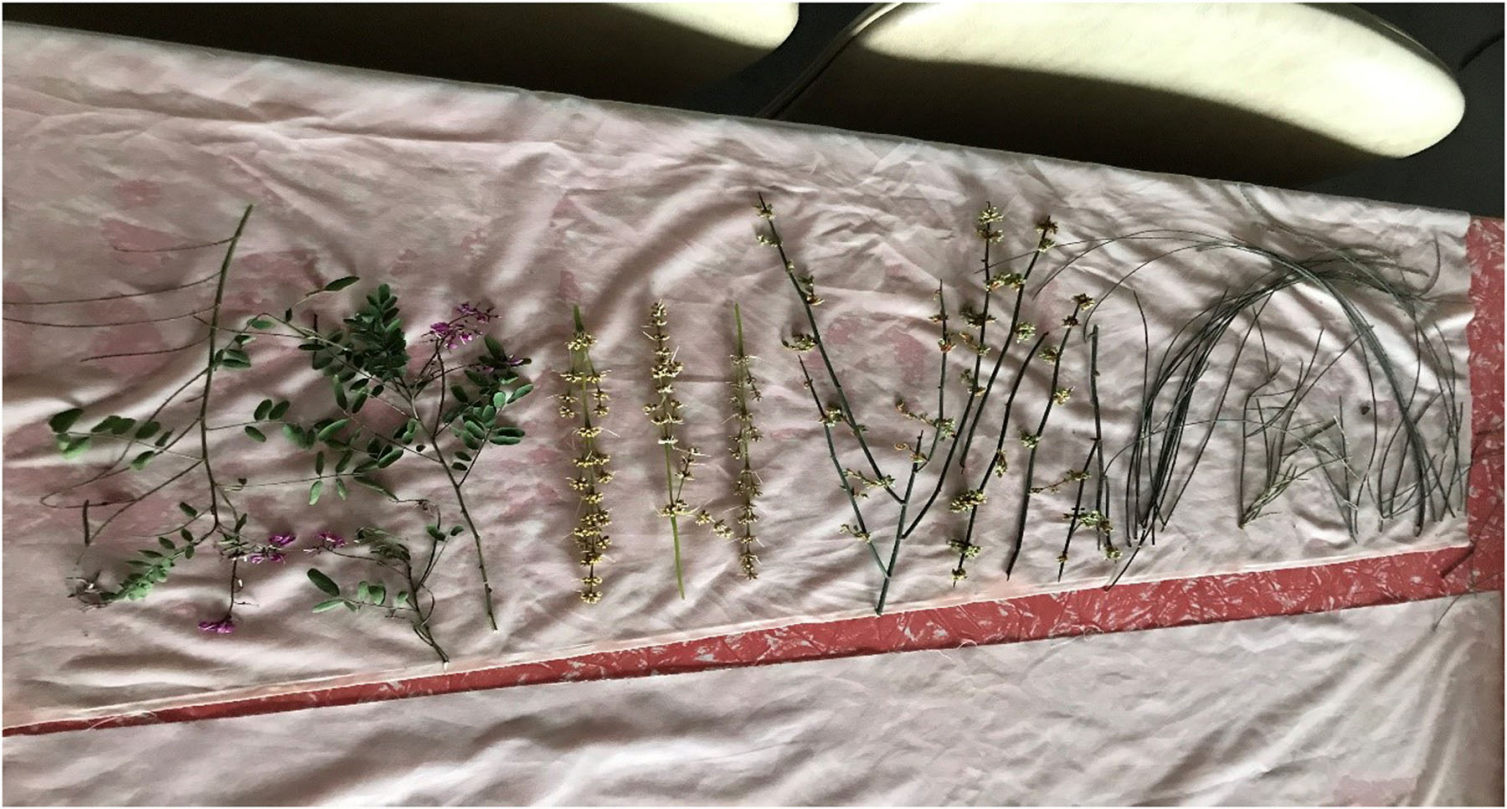
Preparing the healing cloth.

Through a messenger, the women checked in with one another during lockdown, shared their bush‐dyeing or basket‐weaving efforts (sometimes with family members), and photos taken in the Country on daily walks or when visiting their hometowns (once lockdown restrictions were eased). The photos were of being in the Country, of local flora and fauna that reflected how the women were mindfully walking in the country, with a renewed sense of appreciation for and relationship to the natural environment around them. Sometimes, they asked questions of the Elder in the group about stories they have heard or appropriate cultural protocol. Video's capturing birdsongs on morning walks, of their pot boiling their bundle for bush dyeing, or of their local river were also shared. Many also shared images of preparing the clothes for dyeing (see Figure 1).

The women occasionally shared images that captured childhood memories of places and sparked the sharing of stories by other women about their childhood and family. It was also a space where the women made plans for the group in the future. For example, ideas they have for when they would finally get together on Country, and what they can do (e.g., put all the native plants they had used for bush dyeing in a fire). They shared poems and links to articles/resources connected to Aboriginal women, culture, and country. For example, links to information about Wurundjeri calendar of seasons, Aboriginal women's dance, plant identification, Eels feeding, and writing by Aboriginal women writers (including one article that featured a poem from a member of the research team—“In Australia White people write my culture for me”). Sometimes they shared links/images of quotes or personal aspirations that spoke to them in light of yarning that took place during the previous sessions. For example, following a session where the focus was on the role of Aboriginal women (the gatherer), one of the women shared an image her daughter had sent her about Black Matriarchs. That the group valued this space was reflected in comments by group members who sometimes remarked on how they were appreciative of the women sharing those images, for example, if they were having a bad day. Comments also highlighted that this was experienced as a culturally safe space, a homeplace, where they were affirmed by the other women: “it has been a privilege to sit and hold some space with all of you beautiful women. You all certainly have made my world a brighter place to be” (Sarah).

## DISCUSSION

The project reported in this article created settings for Aboriginal women in Melbourne's west who have expressed the need for culturally safe spaces. The research explored how, through Aboriginal cultural practice, Aboriginal women enact healing together. Many of the women who participated are from Aboriginal families with unknown language group identity and unknown connection to Country and at various stages in their identity journeys. Some of them have family who identifies themselves as being part of the Stolen Generations. The workshops were constituted through cultural practices of Wayapa Wuurrk and bush dyeing, which are based on Indigenous Knowledge Systems and conceptions of healing that center Aboriginal spirituality, and ways of being and knowing including connection with Country. Key to the cultural practices is the sharing of cultural knowledge that provides the basis for women's dialogs and recuperation of symbols and practices central to cultural identity and Aboriginal belonging. First Nations belonging is incommensurably different to settler belonging because of Indigenous peoples' ontological relationship to land stemming from the Dreaming, with land understood as constitutive of Aboriginal people rather than as belonging to them (Moreton‐Robinson, 2003). As noted by Moreton‐Robinson, the dislocation caused by policies of removal “means that Indigenous people can be out of place in another's country but through cultural protocols and the commonality of our ontological relationship to country we can be in place but away from our home country” (p. 33).

There is a body of literature that attest to the role of counter and alternative settings in processes of healing, solidarity, and liberation (e.g., Dudgeon & Walker, 2015). Our research concurs with this writing and highlights the value of participation in the dialogs in settings, which influenced the woman in diverse ways. Through intentional dialogs, informed by restorative cultural practices, the women learned and created new knowledge about culture, Country, and self. Through sharing experiences and family histories they connected with the stories of other women establishing intersubjectivity and communality. Through intergenerational storytelling and embodied cultural practices, they reconnected with and reclaimed cultural and spiritual knowledge that has survived colonial violence and attempts at erasure. In line with the writing about the importance of restoring and reclaiming cultural knowledge in liberation processes, this study shows how Aboriginal cosmology and practices can be mobilized to reclaim and strengthen cultural identities (Daher et al., 2020; Dudgeon & Walker, 2015; Fredericks, 2010), especially for those off Country and who were displaced through policies and process that produced the Stolen Generations. The dialogs show how differently positioned women can reconnect and re‐learn ancestral knowledge through cultural practice and connect with Country and culture as part of the process of decolonizing and building social networks.

The cultural practices of Wayapa and bush dyeing and deep listening are central to creating safe healing spaces in which people are supported and nurtured, a humanized space. In line with bell hooks conception of the homeplace, and Case and Hunter's (2012) formulation of counterspaces, the practices mobilized in the setting, affirm Aboriginal knowledge and experience, and provide the mediating tools through which the women supported each other, deconstruct oppression narratives, and affirm Aboriginal identities. The participants experienced the workshops as caring settings in which they were culturally safe. Over the weeks, the women expressed how the space felt intimate and safe. They could be vulnerable and claim vulnerability as empowering because they were with supportive others (see Segalo, 2013). For Atkinson (2002), healing can mean awakening, and reconnecting with community and family, experiencing cultural safety. Importantly, cultural safety “is the identification a person makes with factors derived from the culture, belief systems or worldviews that allow them to feel safe while being with those they have gone for help” (p. 193). The workshops were not constructed as helping settings, but as intentional spaces for healing and community making that happen through sharing stories in a mutually supportive setting characterized by Aboriginal practices of reciprocity, sharing, rooted in knowledge and spiritual understandings of place and Country. Fredericks (2010) in Australia and (Gone, 2013; Gone et al., 2017) in the United States have written about the importance of approaches that allow for the creative and intentional reconnection with traditions and cultural practices, which are central to transformations in Indigenous peoples' identities and experiences of healing and wellbeing.

Our project responds to the recommendations of the Aboriginal and Torres Strait Islander Healing Foundation (2017) and a broader call for decolonizing methodologies (Dudgeon et al., 2020). The workshops that we delivered used embodied cultural practice anchored in Aboriginal knowledge, including meanings of Country that reflect the interconnectedness and coconstitution of people, culture, and the natural environment as the enactment of solidarity and resistance. This is the enactment of decolonial futures through ways of knowing, doing, and being that disrupt the dualistic and independence of people and environment characteristic of the Eurocentric and colonizing approaches (Adams et al., 2015). The practices of care and nurturance and the intentional acts of remembering, connecting to Country, and deconstructing coloniality of gender and race are vital to the process of building networks of support and solidarity and affirming the women in their journeys of cultural reconnection. Both Wayapa and bush‐dyeing introduce Aboriginal ways of knowing, using memory work to bring the past into the present, to name pain, resistance, and struggle. The cultural practices are mobilized towards healing through daily acts of repair (Balla, 2020) and they facilitate cultural connection and opportunities for belonging and community.

Our research and praxis respond to the calls for community research and action to advance decoloniality and epistemic justice. Our project is Indigenous‐led and draws on First Nation's needs, aspirations, and knowledge systems. The approach aligns with community‐engaged and participatory research approaches that seek to foster SEWB in and through research and action with communities (Dudgeon et al., 2020; Gone et al., 2017). We have embraced Indigenous worldviews and concepts of land and Country and knowledge practices of yarning in the process of expanding our ecology of knowledge (Santos, 2014; Sonn, 2016) to impact wellbeing. Our project provides an example of where community psychology and Indigenous methodologies are mobilized to create praxis, a situated knowing, that elevates and prioritizes the needs of communities. In doing so, we have recentered local wisdom and ways of doing as key to counter practices of restoration, repair, and healing.
